# 25KDa branched polyethylenimine increases interferon-γ production in natural killer cells via improving translation efficiency

**DOI:** 10.1186/s12964-023-01101-8

**Published:** 2023-05-09

**Authors:** Eun-Su Ko, Seung Hee Choi, Minwook Lee, Kyung-Soon Park

**Affiliations:** Department of Biomedical Science, CHA University, Seongnam-si, Republic of Korea

**Keywords:** 25KDa branched polyethylenimine, Natural killer cell, Calcium, Interferon-γ, Translation efficiency, ERK signaling, mTOR signaling, eIF4E

## Abstract

**Background:**

Ex vivo cultivation is a promising strategy for increasing the number of NK cells and enhancing their antitumor activity prior to clinical application. Recent studies show that stimulation with 25KDa branched polyethylenimine (25KbPEI) generates NK cells with enhanced antitumor activity. To better understand how 25KbPEI primes NK cells, we explored the mechanism underlying increase in production of IFN-γ.

**Methods:**

Chemical priming was performed on NK-92MI cells by incubating them with 5 μg/ml of 25KbPEI. The production of IFN-γ was evaluated by RT-qPCR, ELISA, and Flow cytometry. By evaluating the effect of pharmacological inhibition of ERK/mTOR-eIF4E signaling pathways on IFN-γ translation, the function of these signaling pathways in IFN-γ translation was examined. To comprehend the level of 25KbPEI activity on immune-related components in NK cells, RNA sequencing and proteomics analyses were conducted.

**Results:**

25KbPEI enhances the production of IFN-γ by NK cells without transcriptional activation. Activation of ERK and mTOR signaling pathways was found to be associated with 25KbPEI-mediated calcium influx in NK cells. The activation of ERK/mTOR signaling was linked to the phosphorylation of 4E-BP1, which resulted in the activation of translation initiation complex and subsequent IFN-γ translation. Analysis of RNA sequencing and proteomics data revealed that the activity of 25KbPEI to improve translation efficiency in NK cells could be extended to additional immune-related molecules.

**Conclusions:**

This study provides substantial insight into the process by which 25KbPEI primes NK cells. Our data demonstrated that the 25KbPEI mediated activation of ERK/mTOR signaling and subsequent stimulation of eIF4E is the primary mechanism by which the chemical stimulates translation of IFN-γ in NK cells.

**Video abstract**

**Supplementary Information:**

The online version contains supplementary material available at 10.1186/s12964-023-01101-8.

## Background

Natural killer (NK) cells are innate immune cells whose cytolytic activity eliminates abnormal cells such as tumor cells and virus-infected cells. The association between decreased NK cell activity and increased cancer susceptibility and poor patient survival suggest that NK cells play an essential role in cancer immunosurveillance [12, 15, 22]. Due to their inherent ability to recognize and eliminate cancer cells without prior sensitization, immunotherapy using adoptively transferred NK cells is considered to be a promising treatment for cancer. Various strategies are employed to improve the therapeutic efficacy of NK cells against cancers, including ex vivo pre-conditioning with cytokines and genetic engineering to express chimeric antigen receptors. When NK cells are activated, integrated signals derived from two distinct types of receptors, noted activating and inhibitory receptors, are transmitted to a cascade of intracellular signaling pathways. Activation of these intracellular signals is directly linked to the cytotoxic activity of NK cells either via accumulation of granzyme/perforin or secretion of cytokines [11, 19]. Therefore, strategies that maximize the activities of these intracellular signals are expected to enhance NK cell cytotoxicity against cancer cells, thereby improving the efficacy of NK cell-based therapies.

Extracellular signal-regulated kinases (ERK) 1/2, major subgroups of Mitogen-activated protein kinases (MAPKs), play essential roles in a variety of biological processes, including cell proliferation, oncogenesis, and activation of immune cells [14, 31]. Binding of NK cell activating receptors to their respective ligands triggers propagation of intracellular signals through ERK1/2 protein-mediated phosphorylation cascades, leading to maturation and polarization of secretory granules towards the immune synapse [5, 21].

In addition to ERK1/2 signaling, mammalian target of rapamycin (mTOR) signaling plays essential role in NK cell activity. Akt/mTOR signaling is highly active in reactive NK cells under both steady-state and stimulated conditions, and decreased phosphorylation of Akt/mTOR signaling results in loss of NK cell activity [25]. Furthermore, mTOR is recognized as the molecular rheostat of NK cells, translating activating stimuli into quantitative tuning of responsiveness [2, 25]. Specifically, mTOR mediates the phosphorylation of eukaryotic translation initiation factor 4E-binding protein 1 (4E-BP1), which leads to the formation of translation initiation complex by releasing eukaryotic translation initiation factor 4E (eIF4E), the translation initiation factor [20, 32, 37].

As representative key signaling pathways that regulate diverse biological processes in response to extracellular cues, Ras-ERK and phosphoinositide 3-kinases (PI3K)-mTORC1 signaling pathways are mutually compensatory, and engage in extensive crosstalk to regulate each other either positively or negatively [27]. During cancer pathogenesis for example, the extent of interplay between ERK1/2 and mTOR signaling pathways is associated with cancer progression and metastasis [35]. However, it is unclear whether crosstalk between ERK 1/2 and mTOR signaling is implicated in NK cell activity.

Previously, we reported that 25KDa branched polyethylenimine (25KbPEI) primes NK cells, resulting in enhanced cytotoxicity via stimulation of calcium influx into the cytosol [6]. NK cells primed with 25KbPEI accumulate mature perforin molecules in the absence of target cells, and this phenotype is linked directly linked to calcium influx. Calcium is an essential signaling molecule that orchestrates numerous biological processes by triggering various intracellular signaling cascades. Even though 25KbPEI induces calcium influx and subsequently enhances the antitumor activity of NK cells, the molecular mechanisms by which 25KbPEI-mediated calcium influx enhances NK cell cytotoxicity remain unclear.

Here, we report that 25KbPEI-mediated calcium influx activated the ERK1/2 and mTOR signaling pathways. The activity of these signaling was directly linked to the phosphorylation of eIF4E and subsequent enhancement of translation efficiency of IFN-γ. RNA sequencing and proteomics analyses suggested that the effect of 25KbPEI on ERK/mTOR/eIF4E axis in NK cells might be extended to other immune-related proteins.

## Materials and methods

### Cell culture

The human NK cell line NK-92MI, the human breast cell line MDA-MB231, and the human ovarian cancer cell lines OVCAR3 and SKOV3 were obtained from the American Type Culture Collection (ATCC, Manassas, Virginia). Human ovarian cancer A2780 cells were obtained from European Collection of Authenticated Cell Cultures (ECACC, Salisbury, UK). NK-92MI cells were cultured in Minimum Essential Medium-alpha (Gibco/Life Technologies, Grand Island, New York) supplemented with 2 mM L-glutamine (Gibco/Life Technologies), 0.1 mM beta-mercaptoethanol (Gibco/Life Technologies), 0.02 mM folic acid (Sigma-Aldrich, St. Louis, Missouri), 0.2 mM inositol (Sigma-Aldrich). MDA-MB231 and A2780 cells were cultured in Dulbecco’s Modified Eagle’s Medium (Gibco/Life Technologies) and Roswell Park Memorial Institute 1640 (Gibco/Life Technologies), respectively. SKOV3 cells were cultured in McCoy’s 5A (modified) medium (Gibco/Life Technologies), and OVCAR3 cells were cultured in Roswell Park Memorial Institute 1640 (ATCC modification) medium (Gibco/Life Technologies) supplemented with 0.01 mg/ml bovine insulin (Sigma-Aldrich). All culture media were supplemented with 10–20% heat-inactivated fetal bovine serum (FBS), along with 1% penicillin/streptomycin (Gibco/Life Technologies).

### Chemical reagents

A stock solution of branched 25KDa Polyethylenimine (25KbPEI, 408727, Sigma-Aldrich) was prepared at a concentration of 10 mg/mL. 1 × 10^6^ NK-92MI cells were treated with 5 μg/ml of 25KbPEI for 12 h in order to generate Chem_NK. After being washed twice with Dulbecco’s Phosphate Buffered Saline (DPBS, 14190-250, Gibco/Life Technologies), Chem_NK cells were resuspended in NK cell culture medium.

The MEK inhibitor U0126 (9903S, Cell Signaling Technology, Danvers, MA), the mTOR inhibitor rapamycin (37094, Sigma-Aldrich), the MNK inhibitor CGP57380 (S7421, Selleckchem, Houston, TX), or the eIF4E-eIF4G binding inhibitor 4EGI-1 (HY-19831, MedChemExpress, Princeton, NJ) were added to the culture medium of NK-92MI cells prior to treatment with 25KbPEI. The TRPM2 channel inhibitor, 2-Aminoethyl diphenylborinate (2-APB, D9754, Sigma-Aldrich) was treated with NK cells. To detect intracellular IFN-γ, NK cells were co-treated with 25KbPEI and Brefeldin A (420601, BioLegend, San Diego, CA) to inhibit cytokine secretion. The translation elongation inhibitor, cycloheximide (CHX, C7698, Sigma-Aldrich) was added for the indicated times after treatment with 25KbPEI. For calcium-free conditions, NK cells were cultured in Suspension Minimum Essential Medium (Gibco/Life Technologies) supplemented with 1 mM ethylene glycol-bis(β-aminoethyl ether)-N,N,N′,N′-tetraacetic acid (EGTA) (Gibco/Life Technologies).

### ELISA

NK-92MI cells were treated with 25KbPEI for 12 h, after which time 1 × 10^6^ cells were co-incubated (for 4 h at 37 °C/5% CO_2_) with target A2780, OVCAR3, SKOV3, and MDA-MB231 cancer cells at an effector:target (E:T) ratio of 10:1. Then, the cell culture supernatants were harvested to measure secreted IFN-γ using the Human IFN-γ ELISA Set (555142, BD Biosciences, San Jose, CA). Absorbance was measured in a microplate reader (Biochrome, Berlin, Germany) at a wavelength of 450 nm.

### Quantitative RT-PCR

Total RNA was extracted from cells using Trizol (INV-15596-018, Thermo Fisher Scientific, WALTHAM, MA), and converted into cDNA using the Super Premium Express 1^st^ Strand cDNA Synthesis System (6250-20, LeGene biosciences, San Diego, CA). Quantitative RT-PCR was performed using iQ SYBR Green PCR Master mix (RT500M, Bio-Rad, Hercules, CA). Data were normalized against the housekeeping gene GAPDH. The oligonucleotide primers used are listed in Additional file 1: Table S1.

### Immunoblot analysis

Cells were lysed in cell lysis buffer (9803S, Cell Signaling Technology) supplemented with a protease and phosphatase inhibitor cocktail (87786, Thermo Fisher Scientific). The protein concentration was measured using a Pierce BCA Protein Assay Kit (23228, Thermo Fisher Scientific). Cell lysates were separated in acrylamide gels and transferred to polyvinylidene difluoride membranes (1620177, Bio-Rad). After blocking in bovine serum albumin for 1 h at room temperature, the membrane was incubated overnight at 4 °C with appropriate primary antibodies, followed by incubation for 1 h at room temperature with the secondary antibody. Immunoblots were imaged by enhanced chemiluminescence (ECL). The antibodies used are listed in Additional file 1: Table S2.

### Flow cytometry analysis

NK cells were stained for 20 min (37 °C/5% CO_2_) with 7-AAD (A1310, Thermo Fisher Scientific) to distinguish live and dead cells. Cells were fixed using 1% paraformaldehyde, washed with FACS buffer (0.09% sodium azide and 2% FBS in DPBS), and permeabilizated with FOXP3 Perm buffer (353097, BioLegend) for 20 min at room temperature. Cells were then resuspended, incubated for 30 min at room temperature in the dark with a fluorochrome-conjugated antibody, and washed twice with FACS buffer. Cells were analyzed using a CytoFLEX flow cytometer (Beckman Coulter, Brea, CA) and data were analyzed using CytExpert (Beckman Coulter) and FlowJo software (Treestar Inc, Ashland, Oregon). The antibodies used are listed in Additional file 1: Table S3.

### Transwell assay

The migration activity of NK-92MI cells was analyzed in a 24-well insert Transwell chamber (8.0 μm; 353097, Corning, Newark, NJ). Cancer cell lines A2780, OVCAR3, and SKOV3 were seeded into the bottom chamber in complete medium and incubated for 24 h. NK-92MI cells in serum-free medium and stained with 1 μM cell trace CFSE (C34554, Thermo Fisher Scientific) were loaded into the upper Transwell insert. After 12 h, the number of CFSE-stained cells in the bottom chamber was counted by a Luna cell counter (Logus, Anyang-si, Republic of Korea).

### RNA-seq analysis

For RNA profiling, mRNA libraries were analyzed using mRNA-Seq (Ebiogen, Seoul, Republic of Korea) and ExDEGA software (Excel Based Differentially Expressed Gene Analysis, Ebiogen). Total RNA was isolated from NK cells using Trizol reagent (Thermo Fisher Scientific) and mRNA libraries were constructed using the QuantSeq 3′ mRNA-Seq Library Prep Kit (Lexogen, Vienna, Austria). High-throughput sequencing was performed (single-end 75 sequencing) using NextSeq 550 (Illumna, San Diego, CA). QuantSeq 3′ mRNA-Seq reads were aligned using Bowtie2 [18]. Bowtie2 indices were either generated from genome assembly sequences or representative transcript sequences prior to aligning with the genome and transcriptome. The RNA sequencing (RNA-seq) data reported in this article have been deposited in National Center for Biotechnology Information (NCBI)’s Gene Expression Omnibus (GEO).

### Proteomics analysis

Peptide libraries were analyzed using LC–MS/MS (Ebiogen) and ExDEGA software. Specifically, total cell lysates were prepared in cell lysis buffer (8 M urea/0.1 M Tris–HCl buffer, pH 8.5) containing a protease inhibitor cocktail (Roche Diagnostics, Indianapolis, IN). The digestion step was performed using Filter Aided Sample Preparation on a Microcon 30 K centrifugal filter device (Millipore, Burlington, Massachusetts). Next, 100 μg of protein was reduced with Tris (2-carboxyethyl) phosphine and alkylated with iodoacetic acid. Then, proteins were digested with trypsin (Promega, Madison, Wisconsin) and the resulting peptides were analyzed on an UltiMate 3000 RSLC nano LC system (Thermo Fisher Scientific) coupled to a Q Exactive plus mass spectrometer (Thermo Fisher Scientific). MS/MS raw files were analyzed using Proteome Discoverer™ software (ver. 2.5).

### Statistical analysis

GraphPad Prism (GraphPad Software, San Diego, CA) software version 7 was used for all statistical calculations. The details of the statistical tests performed are indicated in the figure legends. Data are presented as the mean ± standard deviation (SD). Significance was defined as follows: *P < 0.05; **P < 0.01; ***P < 0.001 or ****P < 0.0001. NS, not significant.

## Results

### 25KbPEI enhances the production of IFN-γ by NK cells without transcriptional activation

**Fig. 1:**
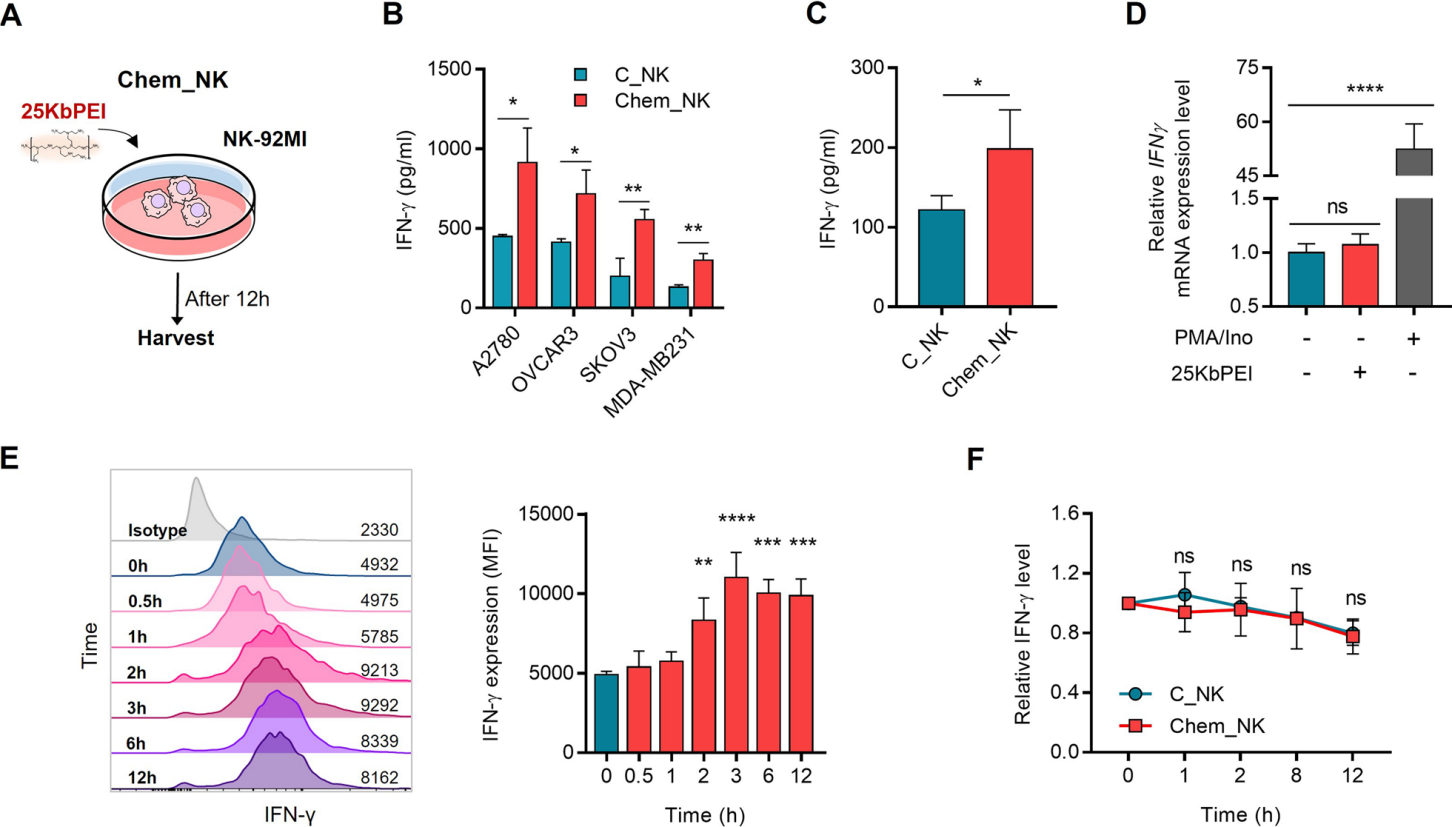
25KbPEI enhances the production of IFN-γ by NK cells without transcriptional activation. **A** Chem_NK cells were generated by treating 1 × 10^6^ NK-92MI cells for 12 h with 5 μg/ml of 25KbPEI. **B** IFN-γ levels present in the co-culture medium of NK cells and target cancer cells was measured by ELISA. **C** IFN-γ levels in culture medium collected from C_NK and Chem_NK cells were measured by ELISA. Statistical analysis was performed using Student’s t-test (vs. C_NK and Chem_NK). **D** *IFN-γ* transcripts in Chem_NK and C_NK cells were measured by qRT-PCR. NK cells treated with PMA/ionomycin were used as a positive control for the analysis of *IFN-γ* transcripts [1]. Statistical analysis was performed using one-way ANOVA with Tukey’s multiple comparisons test (vs. C_NK). **E** Intracellular IFN-γ levels were quantified by flow cytometry after treatment of NK cells with 5 μg/ml 25KbPEI for the indicated times. Statistical analysis was performed using one-way ANOVA with Dunnett’s multiple comparisons test (*vs.* 0 h). **F** Intracellular IFN-γ levels were evaluated by flow cytometry following treatment for 12 h with 25KbPEI and incubation with cycloheximide (CHX) (100 μg/ml) for the indicated times. The IFN-γ level at each time point is represented relative to the level at CHX 0 h. Statistical analysis was conducted using two-way ANOVA with Sidak’s multiple comparisons test (*vs.* C_NK). All experiments were conducted at least three times. *P < 0.05; **P < 0.01; ***P < 0.001, and ****P < 0.0001

Previously, we reported that 25KbPEI enhances the antitumor activity of NK cells [6]. Specifically, NK cells chemically primed with 25KbPEI (Chem_NK) exhibited an enhanced migration activity toward A2780 ovarian cancer cells both in vitro and in vivo [6]. As one of the primary function of IFN-γ is to recruit NK cells to target cancer cells [7, 8, 30], we questioned whether Chem_NK secretes more IFN-γ than control NK cells (C_NK). We generated Chem_NK by treating NK-92MI cells for 12 h with 5 μg/ml 25KbPEI (Fig. 1A). As expected, Chem NK, when co-cultured with ovarian (A2780, OVCAR3, and SKOV3) and triple-negative breast cancer cells (MDA-MB231), released considerably more IFN-γ than C_NK (Fig. 1B). Additionally, IFN-γ production of Chem_NK was higher than that of C_NK in the absence of target cells (Fig. 1C), indicating that 25KbPEI affects the IFN-γ expression machinery in NK cells. Therefore, we investigated the mechanism by which 25KbPEI increases IFN-γ production in NK cells. Treatment with 25KbPEI for 12 h did not increase the number of *IFN-γ* transcripts in NK cells; however, the level of IFN-γ protein began to increase 2 h after exposure to 25KbPEI (Fig. 1D, E). The rate of IFN-γ protein degradation in the presence of the translation elongation inhibitor cycloheximide was comparable between Chem_NK and C_NK cells (Fig. 1F; Additional file 1: Fig. S1), implying that the mechanism by which 25KbPEI increases IFN-γ protein is unrelated to the stability of the IFN-γ protein.

### 25KbPEI increases IFN-γ production by activating ERK/mTOR signaling pathways

**Fig. 2:**
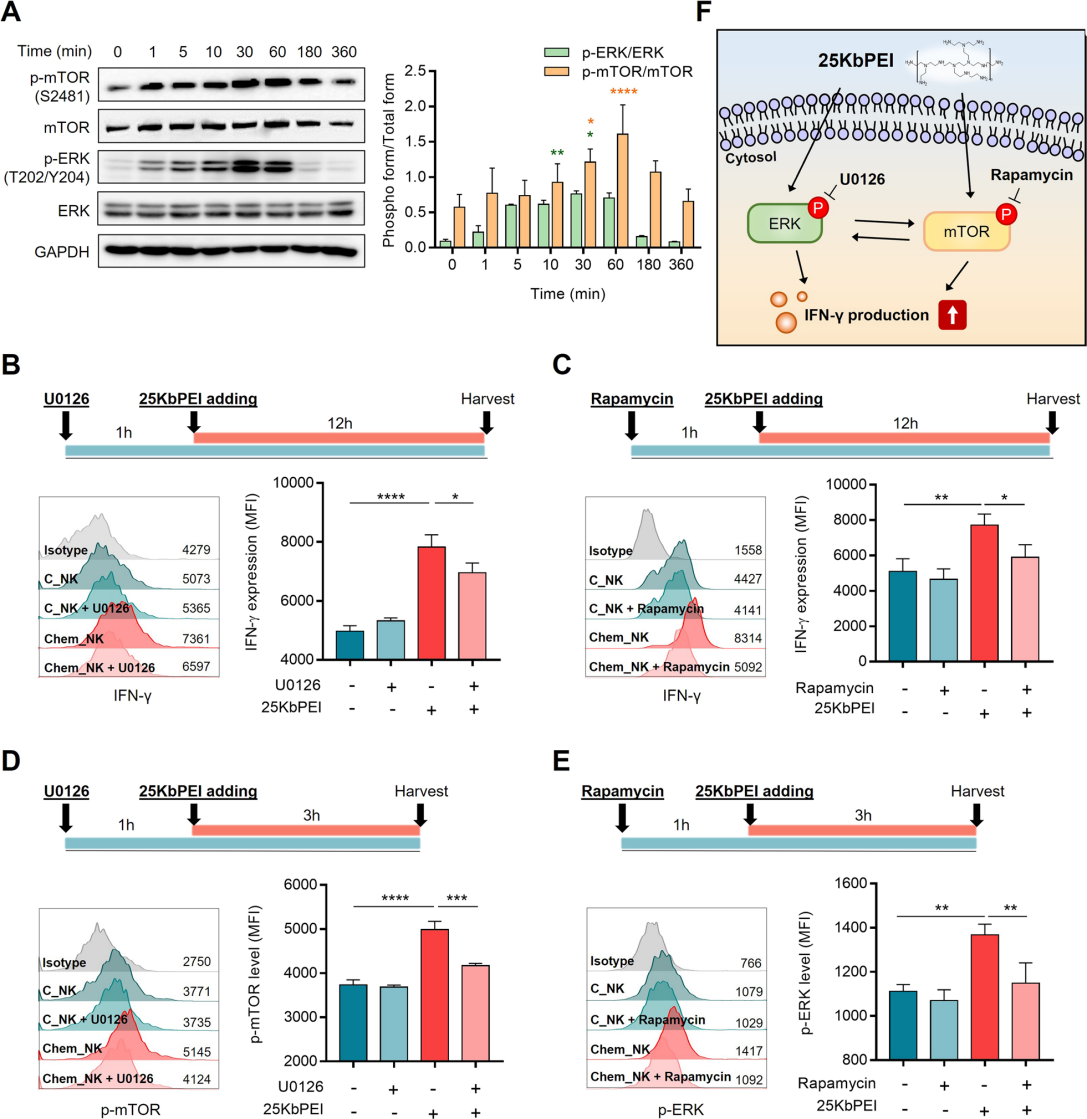
25KbPEI increases IFN-γ production by activating ERK/mTOR signaling pathways. **A** Immunoblot analysis conducted to detect phosphorylation of ERK and mTOR signaling pathway molecules at the indicated times after 25KbPEI treatment of NK-92MI cells (left). The bands were quantified using Image Lab, and the ratio of p-ERK/total ERK or p-mTOR/total mTOR is presented (right). Statistical analysis was conducted using two-way ANOVA with Sidak’s multiple comparisons test (*vs.* 0 h). **B**, **C** NK-92MI cells were pretreated for 1 h with U0126 (20 μM) or rapamycin (50 nM) and then incubated with 25KbPEI for 12 h. IFN-γ levels in the cytosol were measured by flow cytometry. **D**, **E** NK-92MI cells were pretreated with U0126 (20 μM) or rapamycin (50 nM) for 1 h, followed by 25KbPEI for 3 h. Then, p-mTOR or p-ERK levels in NK-92MI cells were measured by flow cytometry. Statistical analysis was conducted using one-way ANOVA with Tukey’s multiple comparisons test. **F** Schematic diagram showing the role of ERK and mTOR signaling in production of IFN-γ by 25KbPEI-treated NK-92MI cells. All experiments were conducted at least three times. *P < 0.05; **P < 0.01; ***P < 0.001, and ****P < 0.0001

Since 25KbPEI began increasing IFN-γ production 2 h after treatment in the absence of transcriptional activation, we investigated whether IFN-γ production is caused by the 25KbPEI-mediated activation of signaling pathways associated with NK cell activity. We noted that ERK and mTOR pathways, which are major signalings in activated NK cells [21, 25, 27], were phosphorylated immediately after 25KbPEI treatment (Fig. 2A).

To confirm whether activation of these two signaling pathways is associated with IFN-γ production, we next examined the effect of chemical inhibition of ERK/mTOR on 25KbPEI-mediated production of IFN-γ. As shown in Fig. 2B, C, the ERK inhibitor U0126 and the mTOR inhibitor rapamycin significantly diminished the effect of 25KbPEI on IFN-γ production (Additional file 1: Fig. S2). These findings suggest that IFN-γ production of Chem_NK is dependent on 25KbPEI-mediated activation of ERK and mTOR signaling pathways. We noted that U0126 inhibited the effect of 25KbPEI on mTOR signaling activation and rapamycin inhibited the effect of 25KbPEI on ERK signaling activation (Fig. 2D, E). Despite the low target specificity of rapamycin [4], our results indicate that 25KbPEI-mediated IFN-γ production may be the result of crosstalk between these two signaling pathways. Taken together, we conclude that 25KbPEI induces IFN-γ production by activating ERK and mTOR signaling pathways (Fig. 2F).

### 25KbPEI-mediated calcium influx induces activation of ERK and mTOR signaling pathways and increases IFN-γ production

Prior to this study, we reported that 25KbPEI stimulates calcium influx into the cytosol of NK cells, which is an essential step for increased perforin accumulation [6]. Calcium is an essential signaling molecule that orchestrates numerous biological processes by triggering intracellular signaling cascades, especially ERK and mTOR pathways, in immune cells [12, 13, 36].

**Fig. 3:**
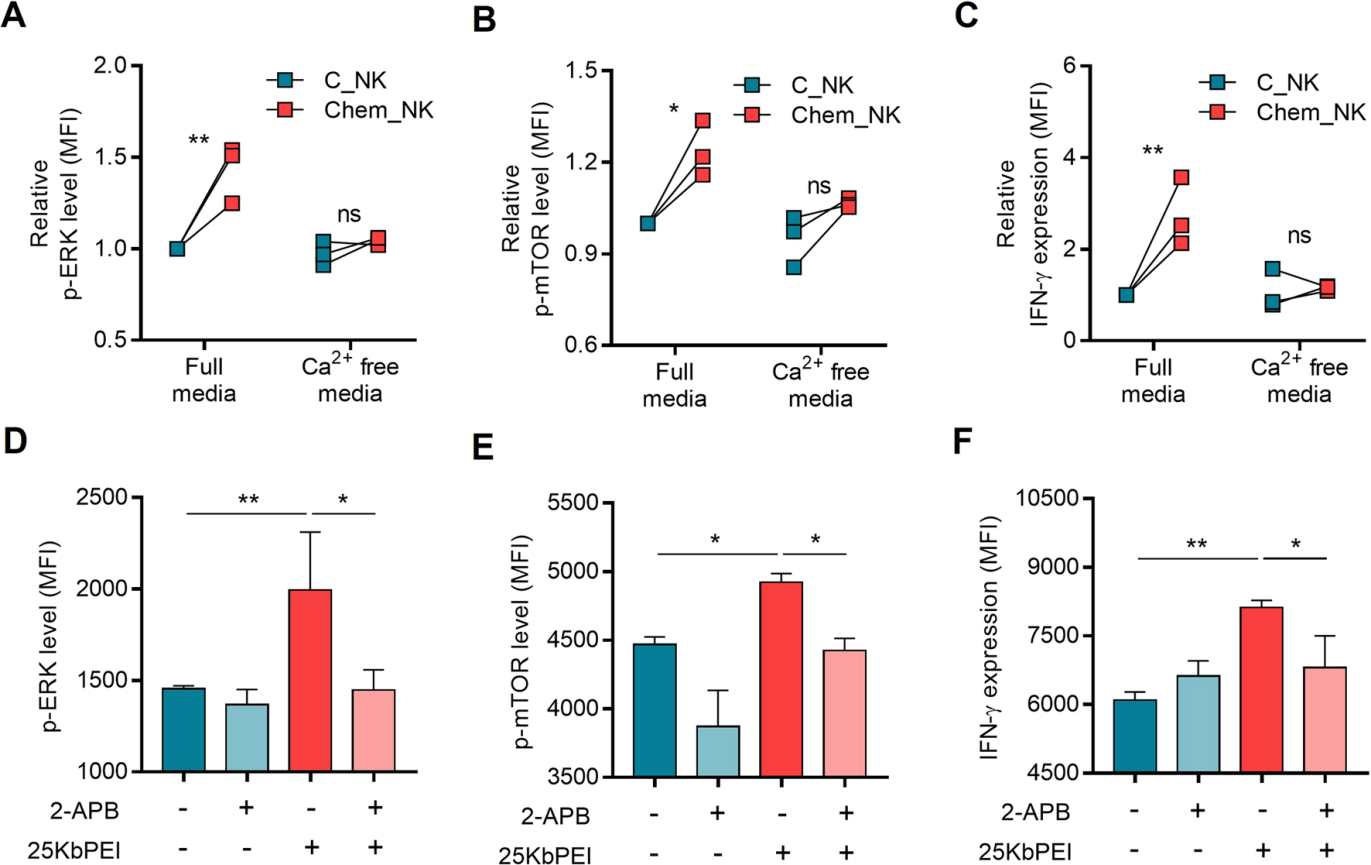
25KbPEI-mediated calcium influx induces activation of ERK and mTOR signaling pathways and increases IFN-γ production. **A**, **B** Phosphorylation of ERK and mTOR in 25KbPEI-treated NK-92MI cells cultured in calcium-free culture medium; p-ERK and p-mTOR were quantified by flow cytometry. **C** IFN-γ production by 25KbPEI-treated NK-92MI cells cultured in calcium-free culture medium; IFN-γ levels in the cytosol were measured by flow cytometry. **D**, **E** Phosphorylation of ERK and mTOR in 25KbPEI-treated NK-92MI cells cultured in the presence of a TRPM2 inhibitor, 2-APB (100 μM). p-ERK and p-mTOR were quantified by flow cytometry. **F** IFN-γ production by 25KbPEI-treated NK-92MI cells cultured in the presence of 2-APB (100 μM). IFN-γ levels in the cytosol were measured by flow cytometry. Statistical analysis was conducted using one-way ANOVA with Tukey’s multiple comparisons test. *P < 0.05; **P < 0.01; ***P < 0.001, and ****P < 0.0001

Therefore, we examined whether 25KbPEI-mediated calcium influx is linked to activation of ERK and mTOR signaling pathways, and to production of IFN-γ, in NK cells. When 25KbPEI was added to NK-92MI cells cultured in calcium-free media, neither ERK nor mTOR were phosphorylated, nor did IFN-γ production increase (Fig. 3A–C). Since TRPM2 is the primary channel responsible for induction of 25KbPEI-mediated calcium influx [6], we next investigated whether chemically inhibiting TRPM2 with 2-APB affects activation of signaling pathways and production of IFN-γ. Treatment with 2-APB blocked the effect of 25KbPEI to phosphorylate ERK and mTOR, and to increase IFN-γ production (Fig. 3D–F). Despite that mTOR signaling of C_NK was also inhibited by 2-APB treatment (Fig. 3E), which may have been due to non-specific effects on other ion channels, such as potassium channels [42], we concluded that 25KbPEI-mediated calcium influx primarily contributes to activation of ERK and mTOR signaling, and to production of IFN-γ.

### 25KbPEI-mediated activation of ERK/mTOR-eIF4E signaling pathways increases the efficiency of IFN-γ translation

**Fig. 4:**
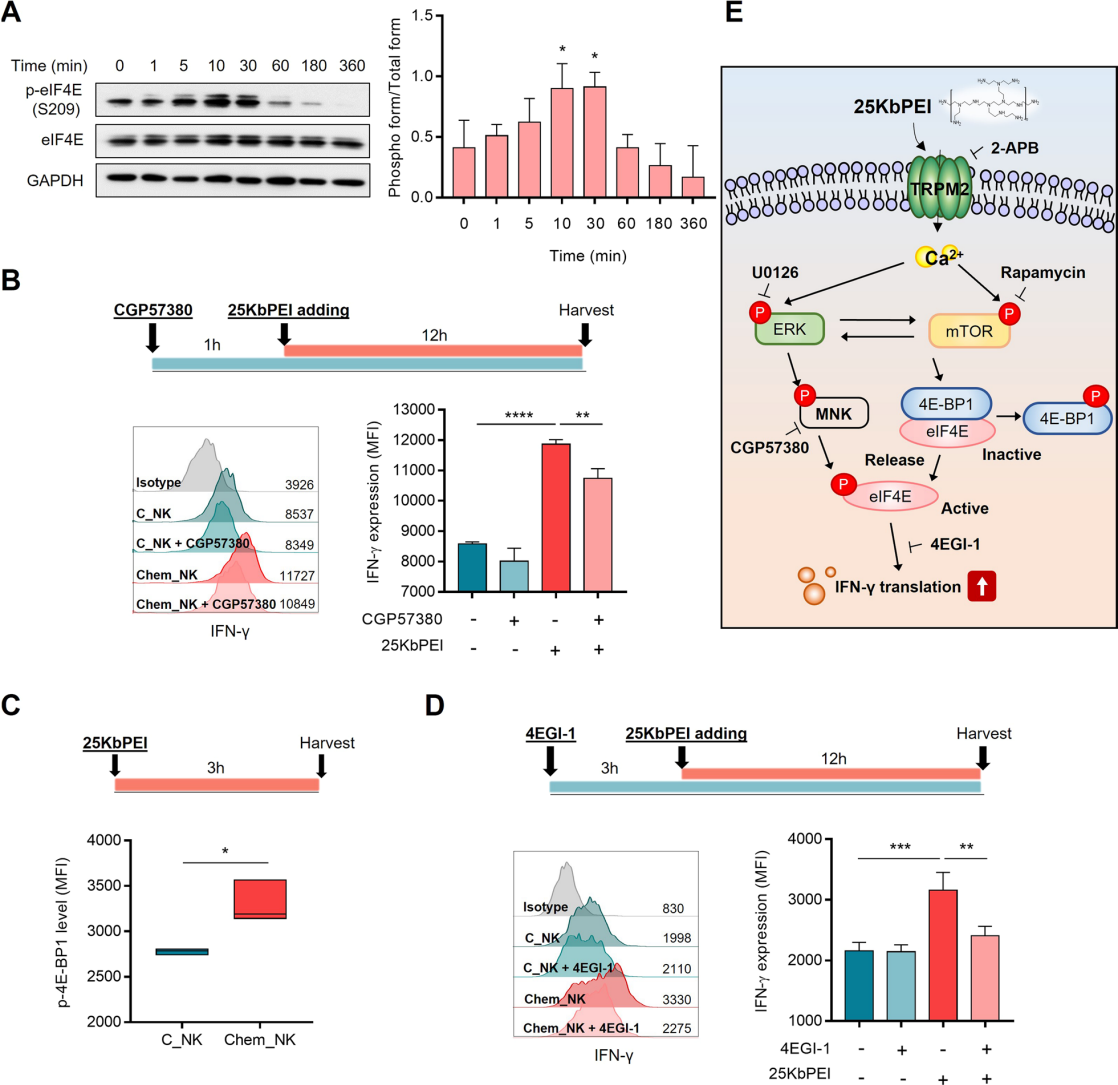
25KbPEI mediated activation of ERK/mTOR-eIF4E signaling pathways increases IFN-γ translation. **A** Immunoblot analysis conducted to detect phosphorylation of eIF4E in NK-92MI cells treated with 25KbPEI for the indicated times (left). The bands were quantified using Image Lab, and the ratio of p-eIF4E/total eIF4E is presented (right). Statistical analysis was performed using one-way ANOVA with Dunnett’s multiple comparisons test (*vs.* 0 h). **B** Amount of IFN-γ in NK-92MI cells treated with 25KbPEI in the presence or absence of CGP57380 (right). NK-92MI cells were pretreated for 1 h with CGP57380 (10 μM) and then incubated with 25KbPEI for 12 h. Cytoplasmic IFN-γ was quantified by flow cytometry. **C** Effect of 25KbPEI on phosphorylation of 4E-BP1 in NK-92MI cells. NK cells were treated with 25KbPEI for 3 h and p-4E-BP1 was quantified by flow cytometry. **D** Amount of IFN-γ in NK-92MI cells treated with 25KbPEI in the presence or absence of 4EGI-1. NK-92MI cells were pretreated for 3 h with 4EGI-1 (50 μM) and then incubated with 25KbPEI for 12 h. Cytoplasmic IFN-γ was quantified by flow cytometry. Statistical analysis was performed using Student's t-test (*vs*. C_NK), and one-way ANOVA with Tukey’s multiple comparisons test. All experiments were conducted at least three times. *P < 0.05; **P < 0.01; ***P < 0.001, and ****P < 0.0001. **E** Schematic diagram showing 25KbPEI-mediated ERK/mTOR signaling, and the role of the downstream factor eIF4E in IFN-γ production by NK-92MI cells treated with 25KbPEI

Since 25KbPEI increases IFN-γ production in the absence of transcriptional activation, we questioned whether 25KbPEI-mediated activation of ERK/mTOR signaling pathways was related to increases in *IFN-γ* mRNA translation. Indeed, it is thought that ERK and mTOR pathways play a central role in regulating the abundance of proteins involved in innate or adaptive immunity [3, 17, 29, 34]. First, we examined the phosphorylation status of eIF4E, considering that translation initiation factor eIF4E is a node for translational control of ‘eIF4E sensitive’ mRNA subsets and eIF4E is activated by phosphorylation [33]. As expected, eIF4E was phosphorylated rapidly by 25KbPEI (Fig. 4A). Chemical inhibition of MNK1, an upstream kinase of eIF4E and a downstream kinase of MAP kinase [38, 41], significantly diminished the effect of 25KbPEI on IFN-γ translation, indicating that 25KbPEI-mediated IFN-γ translation is regulated by ERK signaling-mediated translational control (Fig. 4B; Additional file 1: Fig. S3).

In its non-phosphorylated form, 4E-BP1 interferes with eIF4F complex assembly by disrupting the binding between eIF4E and eIF4G; when phosphorylated by mTORC1, they dissociate, allowing assembly of the active eIF4F complex [10]. 4E-BP1 in Chem_NK cells were phosphorylated significantly more than in C_NK cells (Fig. 4C). In addition to 4E-BP1, ribosomal protein S6 (rpS6), a component of 40S ribosomal protein, was also phosphorylated by mTOR signaling (Additional file 1: Fig. S4). These findings suggest that stimulation of ERK and mTOR signaling by 25KbPEI contributes to activation of eIF4E via distinct pathways.

To further confirm that 25KbPEI-mediated IFN-γ production is induced by translational activation, we examined the effect of inhibiting eIF4E and eIF4G binding to 4EGI-1 on the effect of 25KbPEI to activate IFN-γ translation. In line with other results, treatment with 4EGI-1 significantly diminished the effect of 25KbPEI on IFN-γ translation (Fig. 4D). Taken together, the data suggest that 25KbPEI-mediated activation of ERK/mTOR signaling pathway activates eIF4E to induce translation of IFN-γ (Fig. 4E).

### Inhibition of eIF4E-mediated translation impairs the effect of 25KbPEI on NK cells

**Fig. 5:**
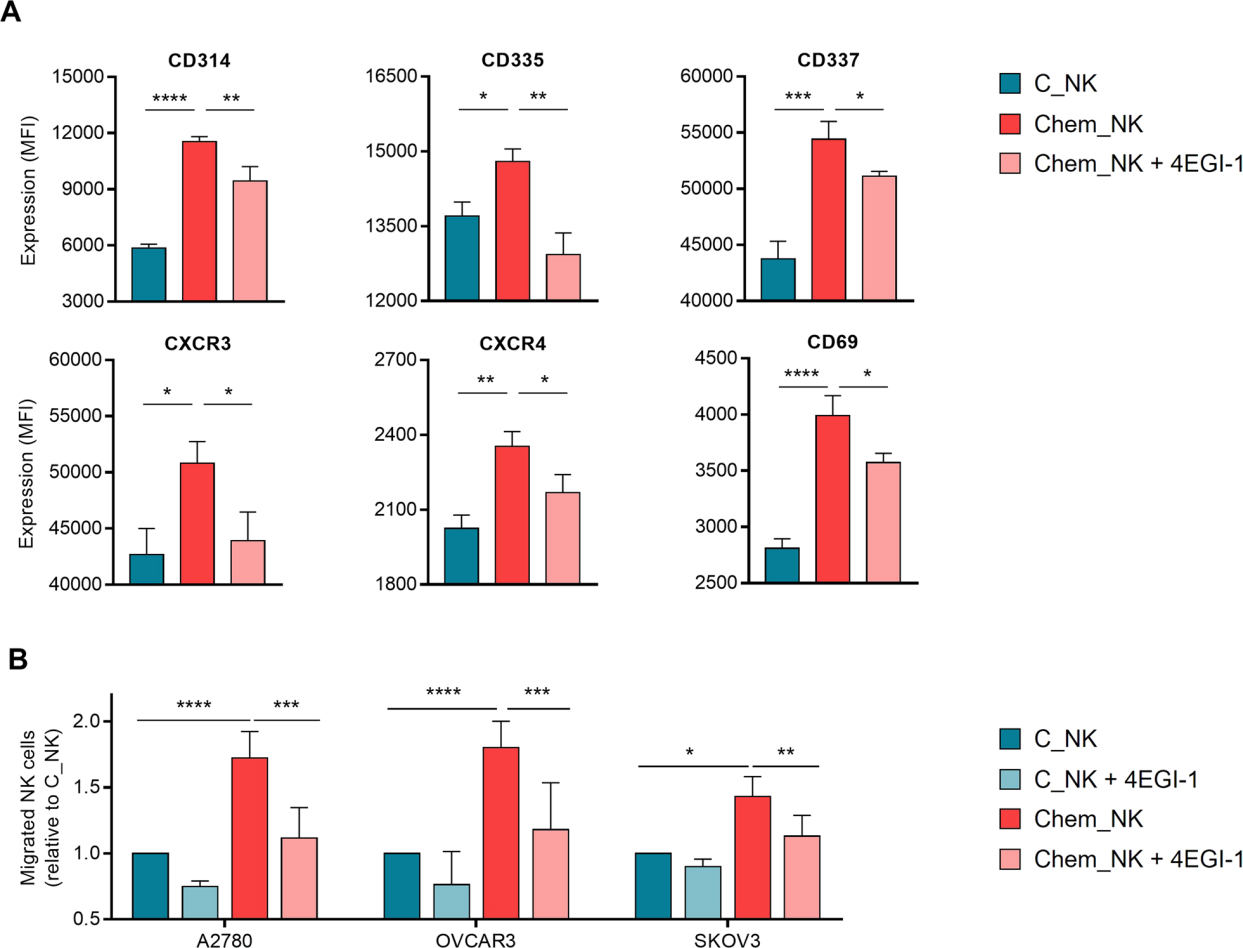
Inhibition of eIF4E-mediated translation impairs the effect of 25KbPEI on NK cells. **A** Expression of the indicated receptors by C_NK and Chem_NK cells was analyzed by flow cytometry. Chem_NK was generated by treating NK-92MI cells with 4EGI-1 (50 μM) for 3 h prior to 25KbPEI treatment. Statistical analysis was conducted using one-way ANOVA with Tukey’s multiple comparisons test. **B** Migration of C_NK and Chem_NK cells toward ovarian cancer cells in the presence or absence of 4EGI-1 (50 μM) was analyzed in Transwell assay. Statistical analysis was conducted using two-way ANOVA with Sidak’s multiple comparisons test. All experiments were conducted at least three times. *P < 0.05; **P < 0.01; ***P < 0.001, and ****P < 0.0001

Previously, we have shown that various membrane receptors associated with immune activation was enhanced in Chem_NK [6]. To determine whether the increase of these receptors is due to eIF4E-dependent enhancement of translation efficiency, mRNA and protein levels of these proteins were analyzed. As expected, the expression of activating and chemokine receptors, which are involved in immune response of NK cells, increased without transcriptional activation; and inhibition of translation initiation complex by 4EGI-1 treatment impaired the accumulation of these proteins in Chem_NK (Fig. 5A; Additional file 1: Fig. S5). Furthermore, the migration activity of Chem_NK have significantly diminished in the presence of 4EGI-1 (Fig. 5B). These findings show that the principal mechanism by which 25KbPEI primes NK cells is the enhancement of eIF4E-mediated translation efficiency of immune-related proteins.

### 25KbPEI stimulates translation of immune related factors in NK cells

**Fig. 6:**
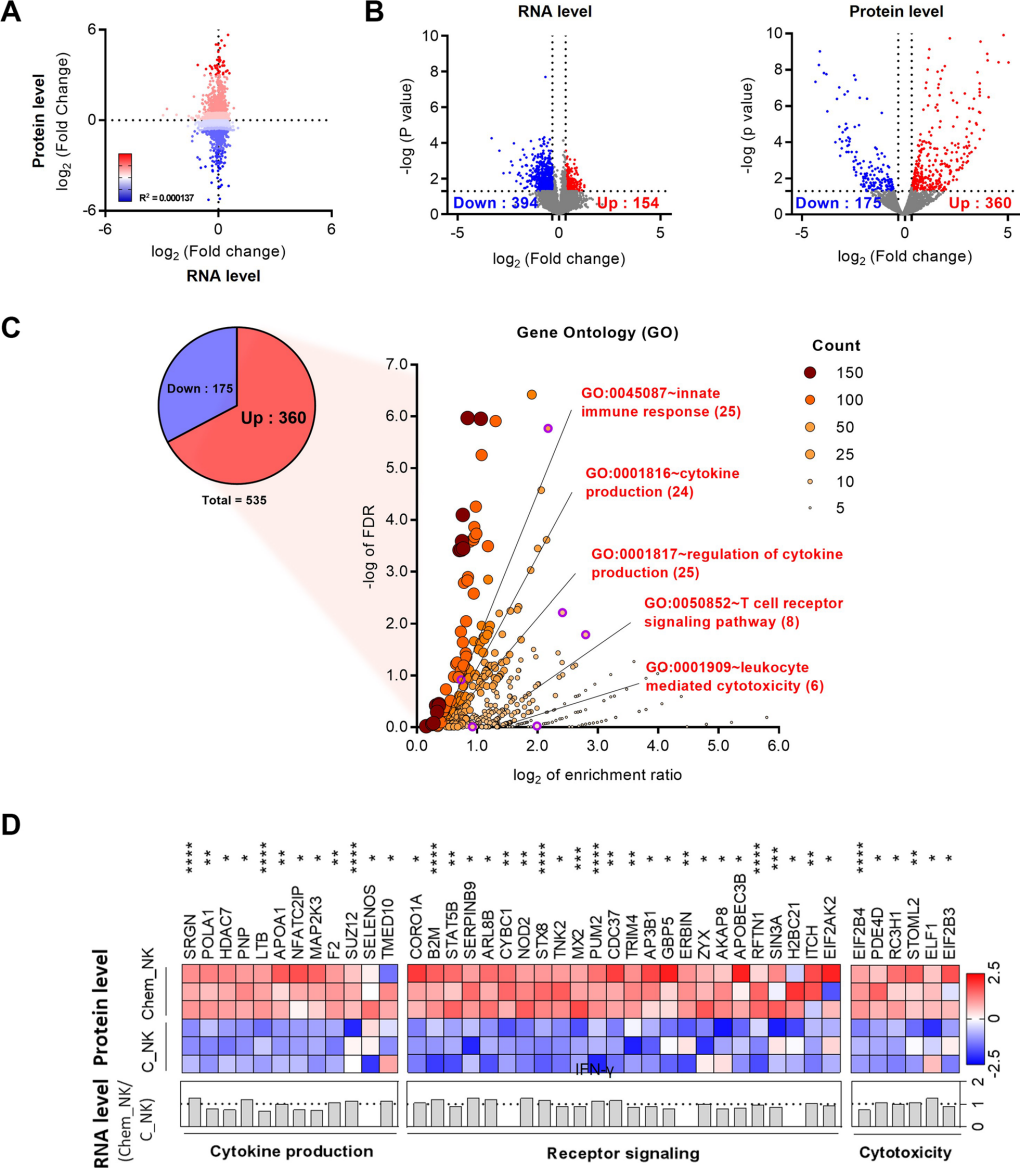
25KbPEI stimulates translation of immune related factors in NK cells. **A** Comparison of -fold changes at the transcriptional (RNA-seq) and translational (Proteomics) levels. **B** Volcano plot of the entire transcriptome (left) or proteome (right) differentially expressed between C_NK and Chem_NK cells. Factors significantly downregulated are shown in blue, and those that are significantly upregulated are shown in red. The vertical lines denote a -fold change of ± 1.25, and the horizontal lines denotes a P value of 0.05. The x-axis represents the log_2_ of the fold change, and the y-axis represents the − log of the P value. **C** Pie graph showing expression of protein products of significantly regulated genes. The dot plots show upregulated Gene ontology (GO) terms enriched in DEGs, and the number of genes enriched within each GO term is represented by dot size and color. Translation-associated GO terms are shown with a purple border. The x-axis represents the log_2_ of the enrichment ratio, and the y-axis represents the − log of the FDR. **D** Heat map of gene expressions associated with immunity GO terms in protein level (upper panel), and in mRNA level (lower panel). The dotted line represents a -fold change of 1. *P < 0.05; **P < 0.01; ***P < 0.001, and ****P < 0.0001

To better understand the extent to which 25KbPEI stimulates translation of proteins, we compared RNA-seq and proteomics data from Chem_NK. As shown in Fig. 6A, the change in protein expression in Chem_NK was more pronounced than that of the transcriptome. Fold-change analysis with a cut-off of 1.25 revealed that ~ 10% (360 out of 3595) of proteins in Chem_NK showed increased expression, whereas only ~ 0.6% (154 out of 25,737) of genes showed increased transcription (Fig. 6B). Notably, Gene Ontology (GO) terms analysis revealed that 42 immunity-associated proteins were included in the ~ 10% of upregulated proteins in Chem_NK cells, whereas R-seq analysis revealed no change in transcription of these genes (Fig. 6C, D). We noted that translation-associated factors in the GO terms included proteins upregulated in Chem_NK, suggesting that 25KbPEI stimulates the overall translation machinery involved in translational efficiency (Fig. 6C; Additional file 1: Fig. S6). This was supported by data showing that the total protein content of Chem_NK was significantly higher than that of C_NK (Additional file 1: Fig. S7). Thus, these findings suggest that increased translation of immunity-related proteins may be the primary mechanism by which 25KbPEI primes NK cells.

## Discussion

The total amount of IFN-γ of a cell is determined by epigenetic, transcriptional, and post-transcriptional factors. With an epigenetically accessible *IFN-γ* locus, mature NK cells show low level constitutive expression of IFN-γ [40]. Until activated by external stimuli such as target cell recognition, NK cells retain pre-transcribed *IFN-γ* mRNAs that are not translated, indicating that pre-formed transcripts are one of the mechanisms by which NK cells rapidly respond to activation [39]. To better understand the response of NK cells to activation stimuli, it is crucial to elucidate the mechanisms underlying translational regulation of IFN-γ.

We showed here that 25KbPEI acts as a priming agent for NK cells to increase levels of immune related proteins such as IFN-γ. Well-known priming reagents include cytokine cocktails that can increase IFN-γ production by increasing the amounts of *IFN-γ* transcripts via activation of transcription factors or by promoting stability of *IFN-γ* mRNA [23, 26, 28]. IL-15 is reported to increase free intracellular calcium in NK cells, which may be used for second messenger signaling to stimulate IFN-γ production [24]. Similarly, we found that 25KbPEI rapidly increased calcium influx, which is then linked to the phosphorylation of ERK/mTOR signaling, eIF4E activation, and increases in IFN-γ production. Considering that ERK1/2 and mTOR signaling primarily affects eIF4E activity to stimulate translation of an ‘eIF4E sensitive’ subset of mRNAs with highly structured 5′ UTRs [43], it would be an interesting question whether 25KbPEI selectively stimulates translation of immune related factors such as *IFN-γ*. In accordance with this prediction, transcriptomic and proteomic analyses indicated that the amounts of numerous immune related proteins increased in 25KbPEI-treated NK cells in the absence of transcriptional activation (Fig. 6D). Moreover, 25KbPEI increased the amount of activating and chemokine receptors, which may contribute to the enhanced migration of NK cells in the absence of transcriptional activation (Fig. 5A, B; Additional file 1: Fig. S5). In fact, proteomic analysis revealed that proteins associated with the translational machinery accumulate in 25KbPEI-treated NK cells, and that the amount of total protein increased in proportion with the duration of 25KbPEI-treatment (Fig. 6C; Additional file 1: Figs. S6 and S7). Our findings indicate that the regulatory mechanism controlling translation of immune related factors may be conserved in NK cells. In addition to eIF4E activity, non-eIF4E mechanisms are also implicated in the functions of immune cells [33]. Since our study focused on 25KbPEI-mediated activation of ERK1/2 and mTOR signaling in NK cells, and since these signaling pathways are involved primarily in eIF4E-dependent translational control, it remains to be investigated whether 25KbPEI-mediated stimulation of intracellular signaling activates non-eIF4E mechanisms to control the translatome in NK cells.

Our study provides significant insight into the relationship between calcium signaling and IFN-γ translation in NK cells. Indeed, several reports show that IFN-γ production by immune cells depends on calcium signaling [9]. For instance, production of IFN-γ by cord blood mononuclear cells requires uptake of extracellular calcium [16]. Moreover, extracellular calcium influx is the initial step in a series of biological processes that occur during NK cell activation upon recognition of target cells. Prior to the current study, we demonstrated that effect of 25KbPEI to enhance the antitumor activity of NK cells is associated primarily with calcium influx through TRMP2, which is directly linked to accumulation of perforin in the absence of target cell recognition [6, 36]. Our study demonstrated that the role of 25KbPEI-mediated calcium influx is not restricted to accumulation of perforin, but also includes eIF4E-dependent translation of numerous immune related factors such as IFN-γ and receptors associated with NK cell activation.

## Conclusions

This study identified calcium influx-mediated phosphorylation of ERK/mTOR and subsequent activation of eIF4E as the primary mechanism by which the chemical induces translation of immune related factors in NK cells. Considering that calcium acts as a second messenger in numerous cell types, including lymphocytes, several additional signaling routes may exist between calcium influx and various biological processes implicated in 25KbPEI-mediated activation of NK cells. Clarifying these pathways would provide new insight into the sequence of events leading to activation of NK cells.

## Supplementary Information

﻿**Additional file 1.** Supplementary figure and tables.

## Data Availability

All relevant data are available from the corresponding author.

## Abbreviations

25KbPEI: 25KDa branched polyethylenimine
4E-BP1: Eukaryotic translation initiation factor 4E-binding protein 1
C_NK: Control NK cells
Ca^2+^: Calcium
Chem_NK: Chemically primed NK cells with 25KbPEI
eIF4E: Eukaryotic translation initiation factor 4E
eIF4F: Eukaryotic initiation factor 4F
eIf4G: Eukaryotic translation initiation factor 4G
ERK: Extracellular signal-regulated kinases
GO: Gene Ontology
IFN-γ: Interferon-gamma
MAPKs: Major subgroups of mitogen-activated protein kinases
MNK: MAPK interacting protein kinase
mTOR: Mammalian target of rapamycin
NK cell: Natural killer cell
TRPM2: Transient receptor potential melastatin 2

## References

[1] Brand A, Singer K, Koehl GE, Kolitzus M, Schoenhammer G, Thiel A, Matos C, Bruss C, Klobuch S, Peter K, Kastenberger M (2016). LDHA-Associated lactic acid production blunts tumor immunosurveillance by T and NK cells. Cell Metab.

[2] Brodin P, Karre K, Hoglund P (2009). NK cell education: not an on-off switch but a tunable rheostat. Trends Immunol.

[3] Brown EJ, Albers MW, Shin TB, Ichikawa K, Keith CT, Lane WS, Schreiber SL (1994). A mammalian protein targeted by G1-arresting rapamycin-receptor complex. Nature.

[4] Carracedo A, Ma L, Teruya-Feldstein J, Rojo F, Salmena L, Alimonti A, Egia A, Sasaki AT, Thomas G, Kozma SC, Papa A (2008). Inhibition of mTORC1 leads to MAPK pathway activation through a PI3K-dependent feedback loop in human cancer. J Clin Invest.

[5] Chen X, Trivedi PP, Ge B, Krzewski K, Strominger JL (2007). Many NK cell receptors activate ERK2 and JNK1 to trigger microtubule organizing center and granule polarization and cytotoxicity. Proc Natl Acad Sci USA.

[6] Choi SH, Kim HJ, Park JD, Ko ES, Lee M, Lee DK, Choi JH, Jang HJ, Kim I, Jung HY, Park KH (2022). Chemical priming of natural killer cells with branched polyethylenimine for cancer immunotherapy. J ImmunoTher Cancer.

[7] Cui F, Qu D, Sun R, Zhang M, Nan K (2020). NK cell-produced IFN-gamma regulates cell growth and apoptosis of colorectal cancer by regulating IL-15. Exp Ther Med.

[8] den Hartog G, Schijf MA, Berbers GAM, van der Klis FRM, Buisman AM (2020). Bordetella pertussis induces IFN-gamma production by NK cells resulting in chemo-attraction by respiratory epithelial cells. J Infect Dis.

[9] Dianzani F, Capobianchi MR, Facchini J (1984). Role of calcium in gamma interferon induction: inhibition by calcium entry blockers. J Virol.

[10] Gingras AC, Raught B, Gygi SP, Niedzwiecka A, Miron M, Burley SK, Polakiewicz RD, Wyslouch-Cieszynska A, Aebersold R, Sonenberg N (2001). Hierarchical phosphorylation of the translation inhibitor 4E-BP1. Genes Dev.

[11] Grund EM, Spyropoulos DD, Watson DK, Muise-Helmericks RC (2005). Interleukins 2 and 15 regulate Ets1 expression via ERK1/2 and MNK1 in human natural killer cells. J Biol Chem.

[12] Guerra N, Tan YX, Joncker NT, Choy A, Gallardo F, Xiong N, Knoblaugh S, Cado D, Greenberg NR, Raulet DH (2008). NKG2D-deficient mice are defective in tumor surveillance in models of spontaneous malignancy. Immunity.

[13] Gulati P, Gaspers LD, Dann SG, Joaquin M, Nobukuni T, Natt F, Kozma SC, Thomas AP, Thomas G (2008). Amino acids activate mTOR complex 1 via Ca^2+^/CaM signaling to hVps34. Cell Metab.

[14] Huang C, Jacobson K, Schaller MD (2004). MAP kinases and cell migration. J Cell Sci.

[15] Huntington ND, Cursons J, Rautela J (2020). The cancer-natural killer cell immunity cycle. Nat Rev Cancer.

[16] Kesson AM, Bryson YJ (1991). Induction of interferon-gamma by cord blood mononuclear cells is calcium dependent. Cell Immunol.

[17] Korneeva NL, Song A, Gram H, Edens MA, Rhoads RE (2016). Inhibition of mitogen-activated protein kinase (MAPK)-interacting kinase (MNK) preferentially affects translation of mRNAs containing Both a 5′-terminal cap and hairpin. J Biol Chem.

[18] Langmead B, Salzberg SL (2012). Fast gapped-read alignment with Bowtie 2. Nat Methods.

[19] Lanier LL (2008). Up on the tightrope: natural killer cell activation and inhibition. Nat Immunol.

[20] Laplante M, Sabatini DM (2012). mTOR signaling in growth control and disease. Cell.

[21] Li C, Ge B, Nicotra M, Stern JN, Kopcow HD, Chen X, Strominger JL (2008). JNK MAP kinase activation is required for MTOC and granule polarization in NKG2D-mediated NK cell cytotoxicity. Proc Natl Acad Sci USA.

[22] Lopez-Soto A, Gonzalez S, Smyth MJ, Galluzzi L (2017). Control of metastasis by NK cells. Cancer Cell.

[23] Lusty E, Poznanski SM, Kwofie K, Mandur TS, Lee DA, Richards CD, Ashkar AA (2017). IL-18/IL-15/IL-12 synergy induces elevated and prolonged IFN-gamma production by ex vivo expanded NK cells which is not due to enhanced STAT4 activation. Mol Immunol.

[24] Luu TT, Schmied L, Nguyen NA, Wiel C, Meinke S, Mohammad DK, Bergö M, Alici E, Kadri N, Ganesan S, Höglund P (2021). Short-term IL-15 priming leaves a long-lasting signalling imprint in mouse NK cells independently of a metabolic switch. Life Sci Alliance.

[25] Marçais A, Marotel M, Degouve S, Koenig A, Fauteux-Daniel S, Drouillard A, Schlums H, Viel S, Besson L, Allatif O, Blery M (2017). High mTOR activity is a hallmark of reactive natural killer cells and amplifies early signaling through activating receptors. Elife.

[26] Mavropoulos A, Sully G, Cope AP, Clark AR (2005). Stabilization of IFN-gamma mRNA by MAPK p38 in IL-12- and IL-18-stimulated human NK cells. Blood.

[27] Mendoza MC, Er EE, Blenis J (2011). The Ras-ERK and PI3K-mTOR pathways: cross-talk and compensation. Trends Biochem Sci.

[28] Nakahira M, Ahn HJ, Park WR, Gao P, Tomura M, Park CS, Hamaoka T, Ohta T, Kurimoto M, Fujiwara H (2002). Synergy of IL-12 and IL-18 for IFN-gamma gene expression: IL-12-induced STAT4 contributes to IFN-gamma promoter activation by up-regulating the binding activity of IL-18-induced activator protein 1. J Immunol.

[29] Nandagopal N, Roux PP (2015). Regulation of global and specific mRNA translation by the mTOR signaling pathway. Translation.

[30] Pak-Wittel MA, Yang L, Sojka DK, Rivenbark JG, Yokoyama WM (2013). Interferon-gamma mediates chemokine-dependent recruitment of natural killer cells during viral infection. Proc Natl Acad Sci USA.

[31] Pearson G, Robinson F, Beers Gibson T, Xu BE, Karandikar M, Berman K, Cobb MH (2001). Mitogen-activated protein (MAP) kinase pathways: regulation and physiological functions. Endocr Rev.

[32] Pelletier J, Graff J, Ruggero D, Sonenberg N (2015). Targeting the eIF4F translation initiation complex: a critical nexus for cancer development. Cancer Res.

[33] Piccirillo CA, Bjur E, Topisirovic I, Sonenberg N, Larsson O (2014). Translational control of immune responses: from transcripts to translatomes. Nat Immunol.

[34] Sabatini DM, Erdjument-Bromage H, Lui M, Tempst P, Snyder SH (1994). RAFT1: a mammalian protein that binds to FKBP12 in a rapamycin-dependent fashion and is homologous to yeast TORs. Cell.

[35] Saini KS, Loi S, de Azambuja E, Metzger-Filho O, Saini ML, Ignatiadis M, Dancey JE, Piccart-Gebhart MJ (2013). Targeting the PI3K/AKT/mTOR and Raf/MEK/ERK pathways in the treatment of breast cancer. Cancer Treat Rev.

[36] Schwarz EC, Qu B, Hoth M (2013). Calcium, cancer and killing: the role of calcium in killing cancer cells by cytotoxic T lymphocytes and natural killer cells. Biochim Biophys Acta.

[37] So L, Lee J, Palafox M, Mallya S, Woxland CG, Arguello M, Truitt ML, Sonenberg N, Ruggero D, Fruman DA (2016). The 4E-BP-eIF4E axis promotes rapamycin-sensitive growth and proliferation in lymphocytes. Sci Signal.

[38] Sonenberg N, Gingras AC (1998). The mRNA 5′ cap-binding protein eIF4E and control of cell growth. Curr Opin Cell Biol.

[39] Stetson DB, Mohrs M, Reinhardt RL, Baron JL, Wang ZE, Gapin L, Kronenberg M, Locksley RM (2003). Constitutive cytokine mRNAs mark natural killer (NK) and NK T cells poised for rapid effector function. J Exp Med.

[40] Tato CM, Martins GA, High FA, DiCioccio CB, Reiner SL, Hunter CA (2004). Cutting Edge: Innate production of IFN-gamma by NK cells is independent of epigenetic modification of the IFN-gamma promoter. J Immunol.

[41] Waskiewicz AJ, Flynn A, Proud CG, Cooper JA (1997). Mitogen-activated protein kinases activate the serine/threonine kinases Mnk1 and Mnk2. EMBO J.

[42] Zhuo RG, Liu XY, Zhang SZ, Wei XL, Zheng JQ, Xu JP, Ma XY (2015). Insights into the stimulatory mechanism of 2-aminoethoxydiphenyl borate on TREK-2 potassium channel. Neuroscience.

[43] Zimmer SG, DeBenedetti A, Graff JR (2000). Translational control of malignancy: the mRNA cap-binding protein, eIF-4E, as a central regulator of tumor formation, growth, invasion and metastasis. Anticancer Res.

