# Supplementary material for: 25KDa branched polyethylenimine increases interferon-γ production in natural killer cells via improving translation efficiency
Source: Cell Commun Signal. 2023 May 9;21:107. doi: 10.1186/s12964-023-01101-8 (PMC10170831; doi:10.1186/s12964-023-01101-8)
Supplement: Supplementary file 2 — ﻿Additional file 1. Supplementary figure and tables. [file 12964_2023_1101_MOESM2_ESM.docx]

**25KDa branched polyethylenimine increases interferon-γ production in natural killer cells via improving translation efficiency**

*Eun-Su Ko^†^, Seung Hee Choi,^†^, Minwook Lee, and Kyung-Soon Park^*^*

Department of Biomedical Science, CHA University, Seongnam, Republic of Korea

^†^These authors equally contributed to this work.

***Corresponding authors**

kspark@cha.ac.kr (K.-S.P.)

**Key Words**

25KDa branched polyethylenimine, Natural killer cell, Calcium, Interferon-γ, Translation efficiency, ERK signaling, mTOR signaling, eIF4E

**Supplemental information**

**
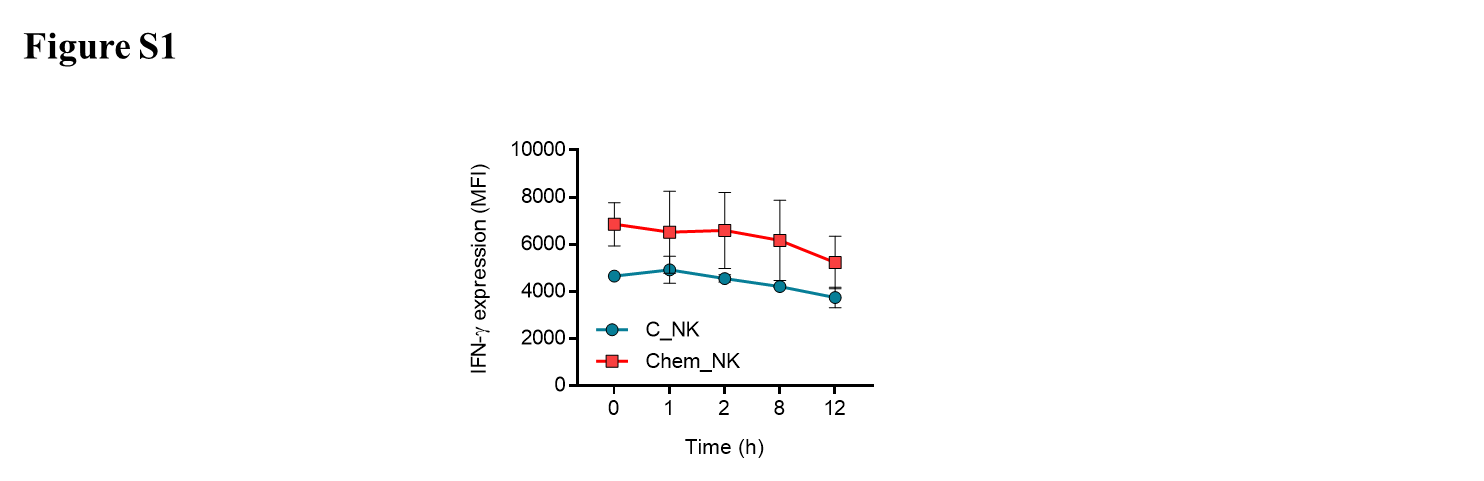
**

**Figure S1.** Intracellular IFN-γ levels were evaluated by flow cytometry following treatment for 12 h with 25KbPEI and incubation with cycloheximide (CHX) (100 μg/ml) for the indicated times.


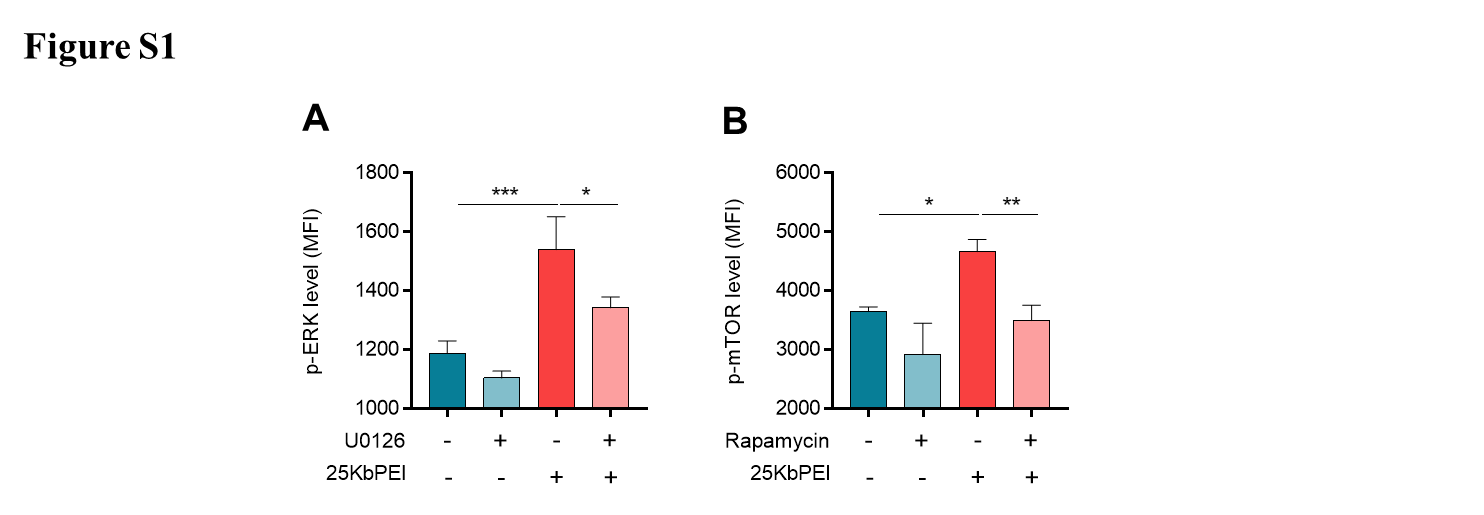


**Figure S2. (A)** NK-92MI cells were pretreated for 1 h with U0126 (20 μM), followed by 25KbPEI for 3 h; p-ERK levels were measured by flow cytometry. (**B)** NK-92MI cells were pretreated for 1 h with rapamycin (50 nM), followed by 25KbPEI for 3 h; p-mTOR levels were measured by flow cytometry. Statistical analysis was conducted using one-way ANOVA with Tukey’s multiple comparisons test. *P < 0.05, **P < 0.01, and ***P < 0.001.

**
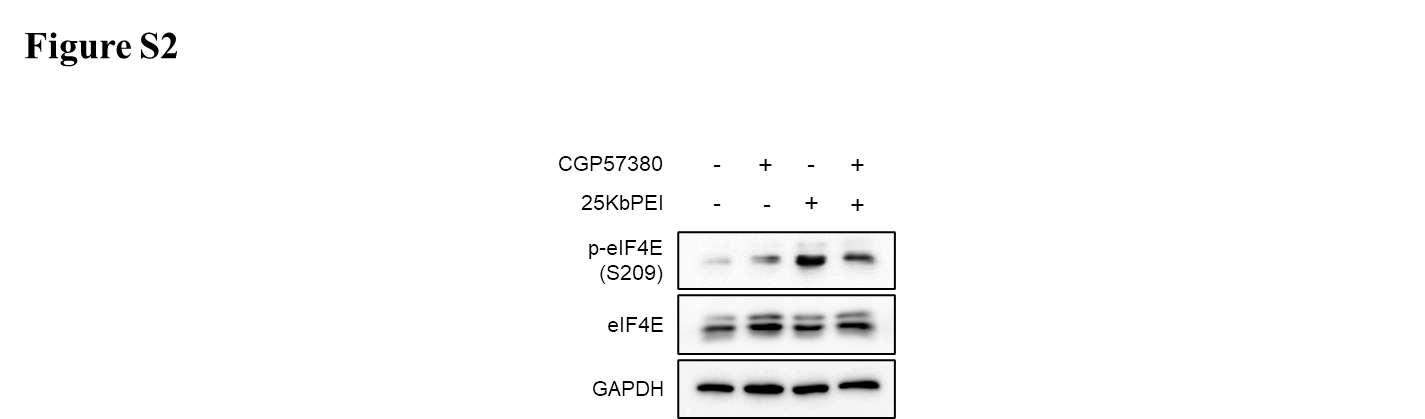
**

**Figure S3.** NK-92MI cells were incubated for 1 h with CGP57380 (10 μM) and then treated with 25KbPEI. p-eIF4E and eIF4E levels were measured by western blotting.


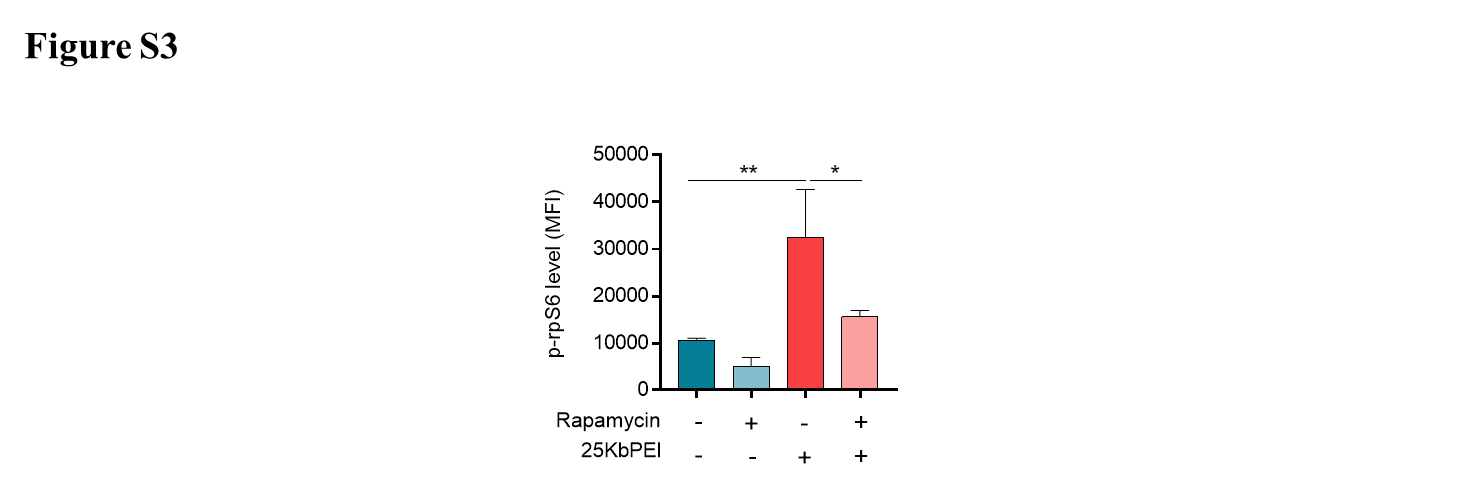


**Figure S4.** NK-92MI cells were pretreated for 1 h with rapamycin (50 nM), followed by 25KbPEI for 3 h; p-rpS6 levels were measured by flow cytometry. Statistical analysis was conducted using one-way ANOVA with Tukey’s multiple comparisons test. The experiment was conducted at least three times. *P < 0.05, and **P < 0.01.


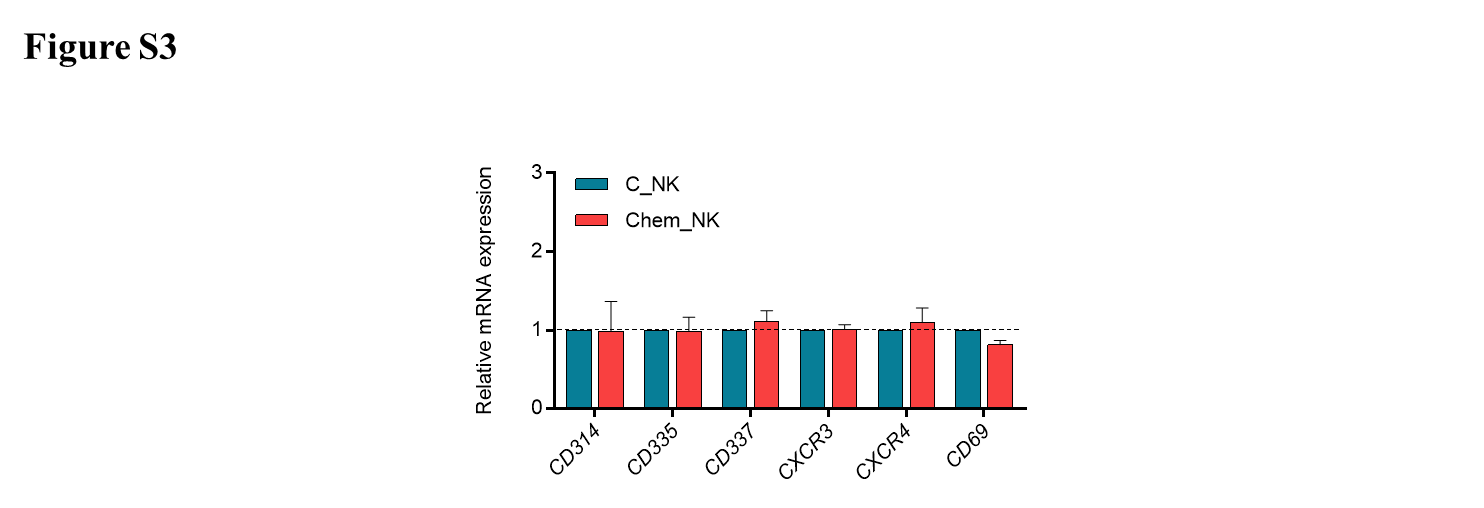


**Figure S5.** Expression of mRNA encoding the indicated receptors was measured by qRT-PCR. Statistical analysis was performed using Student's t-test (*vs.* C_NK and Chem_NK). All experiments were conducted at least three times.

**
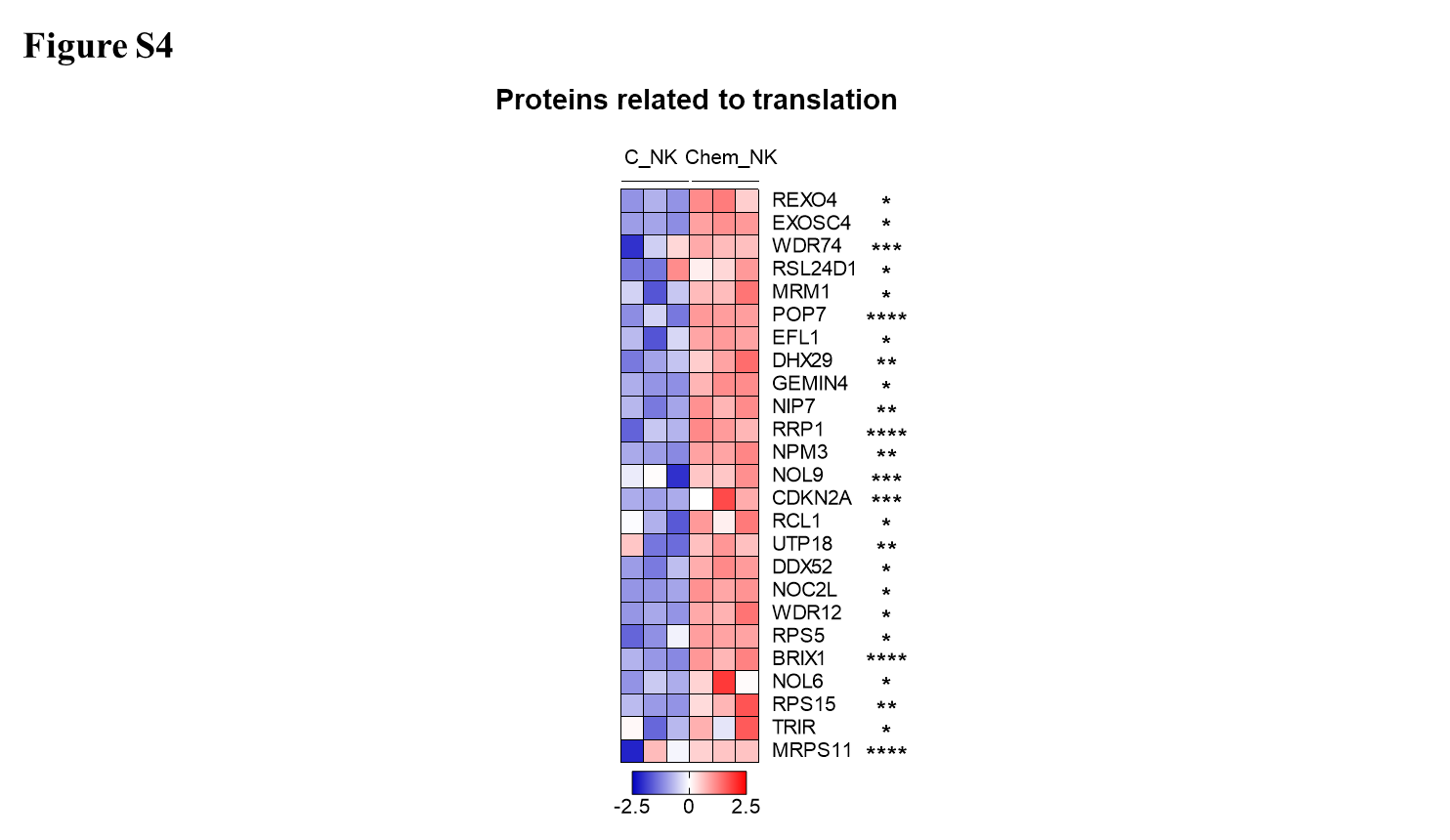
**

**Figure S6.** Comparison of genes associated with translation GO terms in the proteomics heat map. *P < 0.05, **P < 0.01, ***P < 0.001, and ****P < 0.0001.

**
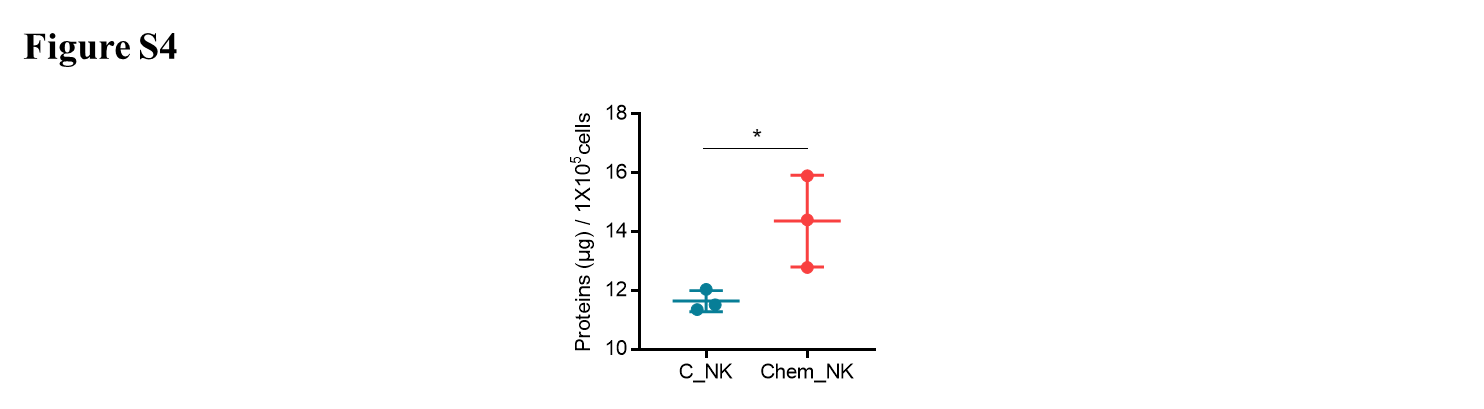
**

**Figure S7.** Fold changes in total proteins in Chem_NK compared with C_NK. The protein content of 1×10^5^ cells treated with 5 μg/ml 25KbPEI for 12 h was measured in a BCA assay. Statistical analysis was performed using Student's t-test (*vs*. C_NK). The experiment was conducted in triplicate. *P < 0.05, **P < 0.01, ***P < 0.001, and ****P < 0.0001.

**Supplemental table 1. Primers**

| **Gene name** | **Forward (5' to 3')** | **Reverse (5' to 3')** |
| --- | --- | --- |
| *CD314* | CAAGATCTTCCCTCTCTGAGCA | TGCTTTTGCCATCGTGTTGAA |
| *CD335* | CTAGGCCGGCAGAATCTG | CCTGGCAACAGATGGTCACT |
| *CD337* | CTTGGCCTCCTSGAGACCCT | TTGTAGCCAGGCCTTTGGTC |
| *CXCR3* | ACCACCAAGTGCTAAATGAC | CAGAAAGAGGAGGCTGTAGA |
| *CXCR4* | GCATGACGGACAAGTACAGGCT | AAAGTACCAGTTTGCCACGGC |
| *CD69* | GAGCTGGACTTCAGCCCAAA | CCACTTCCATGGGTGACCAG |
| *IFN-γ* | ACTGTCGCCAGCAGCTAAAA | TATTGCAGGCAGGACAACCA |
| *APDH* | GGGTGTGAACCATGAGAA | GTCTTCTGGGTGGCAGTGAT |

**Supplemental table 2. Antibodies for immunoblot.**

| **Antibody** | **Vendor** | **Cat.** |
| --- | --- | --- |
| mTOR | Cell Signaling | 2983P |
| p-mTOR (S2481) | Cell Signaling | 2974P |
| ERK1/2 | Cell Signaling | 4695S |
| p-ERK1/2 (T202/Y204) | Cell Signaling | 4370L |
| eIF4E | Cell Signaling | 9742L |
| p-eIF4E (S209) | Cell Signaling | 9741L |
| HRP-conjugated GAPDH | Cell Signaling | 3683S |
| Goat Anti-Rabbit IgG antibody (HRP) | Gene Tex | GTX213110-01 |

**Supplemental table 3. Antibodies for flow cytometry.**

| **Antibody** | **Fluorochrome** | **Vendor** | **Cat.** |
| --- | --- | --- | --- |
| CD314 | PE | BD | 557940 |
| CD335 | PE | BD | 557991 |
| CD337 | PE | BD | 325207 |
| IFN-γ | PE | BD | 554701 |
| p-mTOR (S2448) | PE | BD | 563489 |
| Isotype control | PE | BD | 551436 |
| CD69 | PerCP | BioLegend | 310927 |
| CXCR3 | PC Cy5.5 | BioLegend | 353714 |
| CXCR4 | PC Cy5.5 | BioLegend | 306515 |
| Isotype control | PC Cy5.5 | eBioscience | 45-4031-80 |
| p4E-BP1 (T37/46) | Alexa647 | Cell Signaling | 5123S |
| p-ERK (T202/Y204) | APC | BioLegend | 369522 |
| Isotype control | APC | BD | 551414 |
